# A suspicious dark lesion in a boy

**DOI:** 10.1002/ccr3.2171

**Published:** 2019-04-29

**Authors:** Vera Tengattini, Michelangelo La Placa, Iria Neri, Silvia Martini, Annalisa Patrizi

**Affiliations:** ^1^ Division of Dermatology, Department of Experimental, Diagnostic and Specialty Medicine University of Bologna Bologna Italy; ^2^ Department of Medical and Surgical Sciences University of Bologna Bologna Italy

**Keywords:** atypical, crust, dermatosis, nevus

## Abstract

The presence of history of hyperpigmented crust in a patient with a history of adequate hygiene is typical of terra firma‐forme dermatosis. The treatment is the rubbing of the skin with isopropyl alcohol (removal of the hyperpigmented brown crust, confirming the diagnosis). Physicians should be aware because early diagnosis avoid unnecessary treatment.

## 1 INTRODUCTION

**Figure 1:**
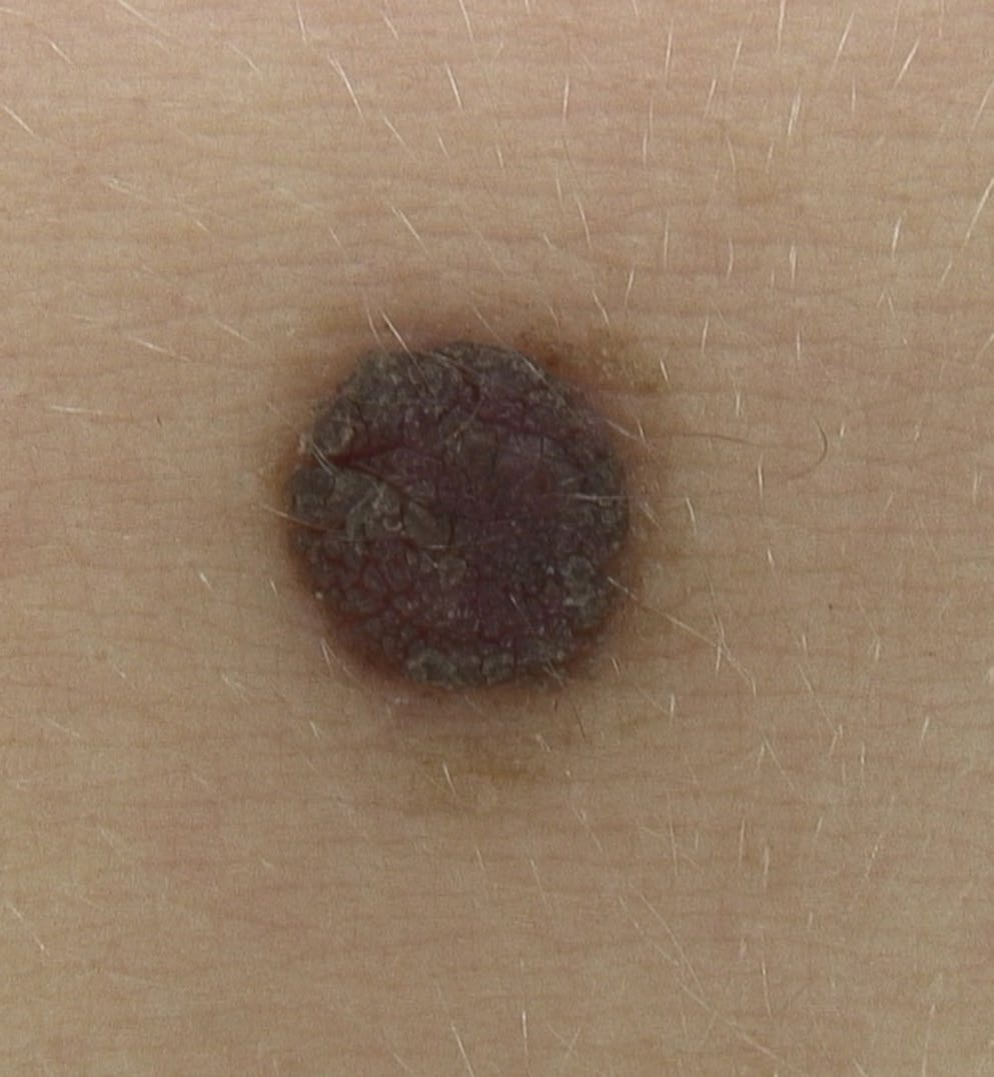
Nonpruritic nevus covered by fine hyperpigmented crust on the abdominal region

A 15‐year‐old boy presented with a nonpruritic nevus covered by fine hyperpigmented crust on his abdominal region (Figure 1). His parents reported that the clinical modification had appeared 1 year ago. Total body examination of the skin did not show other similar lesions.

What would you do next?

- Remove the hyperpigmented crust
- Follow‐up
- Topical steroid
- Program a biopsy

## 2 DISCUSSION AND OUTCOMES

**Figure 2:**
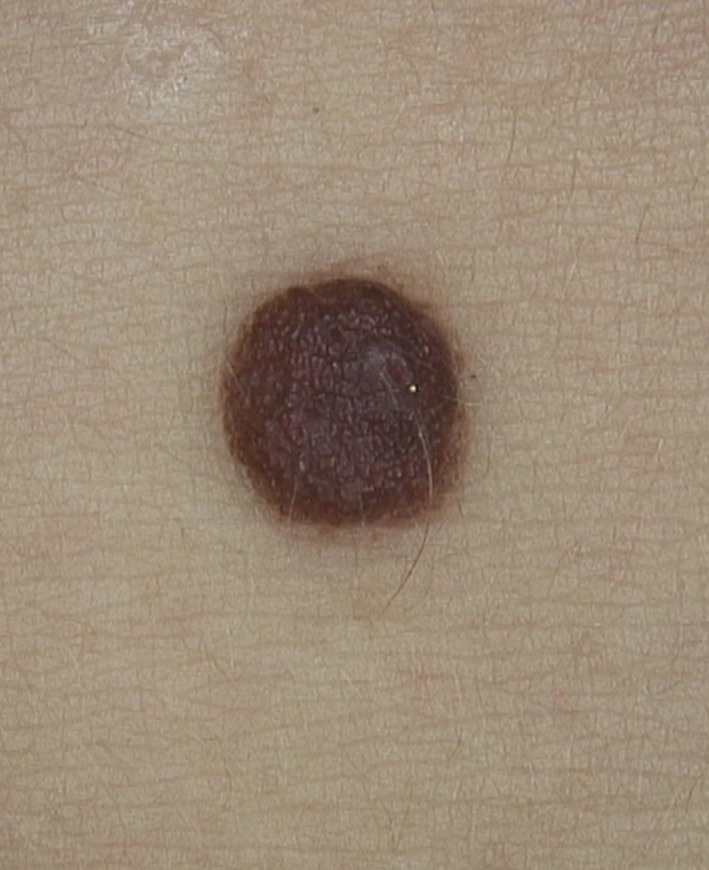
The rubbing of the skin with isopropyl alcohol achieved the removal of the halo, revealing a benign dermal nevus

The key clinical feature of this case is the presence of an hyperpigmented crust in a patient with a history of adequate hygiene. The rubbing of the skin with isopropyl alcohol achieved the removal of the hyperpigmented brown crust, confirming the diagnosis of terra firma‐forme dermatosis (TFFD)^1^ and revealing a benign dermal nevus (Figure 2).

Terra firma‐forme dermatosis is a clinical condition first described by Duncan in 1987^2^ due to disordered keratinocyte buildup and compaction with surrounding sebum and dirt.^3^ Hyperpigmented, brown‐grayish patches and plaques characterize the clinical presentation of TFFD (presenting as dirt‐like plaques, hence the name from Latin) more frequently involving the face, trunk, neck, and the ankles.^1^ Application and accumulation of residues of soaps, emollients on dry skin may also contribute to its formation.^1^ The gold standard test is isopropyl alcohol swab and treatment with keratolytic agents such urea emollients.^1^ When patients and parents are educated regarding the condition, no recurrence usually occurs.

Terra firma‐forme dermatosis is often misdiagnosed (differential diagnosis included verrucous epidermal nevi, pityriasis versicolor, dermatitis neglecta etc^1^, ^2^, ^3^ tinea versicolor, ashy dermatosis, atopic dermatitis…): in our patient, other physicians had misinterpreted this phenomenon as an atypical nevus and we avoided unnecessary surgical removal.

## CONFLICT OF INTEREST

The authors have no conflict of interest to disclose.

## AUTHOR CONTRIBUTION

VT, MLP, AP, SM, IN: All authors have participated in the work: (a) substantial contributions to conception and design, acquisition of data, or analysis and interpretation of data; (b) drafting the article or revising it critically for important intellectual content; and (c) final approval of the version to be published.
